# Natural and Pathological Autoantibodies Show Age-Related Changes in a Spontaneous Autoimmune Mouse (NZB) Model

**DOI:** 10.3390/ijms24129809

**Published:** 2023-06-06

**Authors:** Szonja Gál, Erzsébet Gajdócsi, Esam Khanfar, Katalin Olasz, Diána Simon, Péter Balogh, Tímea Berki, Péter Németh, Ferenc Boldizsár

**Affiliations:** 1 Department of Immunology and Biotechnology, Medical School, University of Pecs, H-7624 Pécs, Hungary; gal.patricia.szonja@gmail.com (S.G.); gajdocsi.erzsebet@pte.hu (E.G.); esam.khanfar@pte.hu (E.K.); olasz.katalin@pte.hu (K.O.); simon.diana@pte.hu (D.S.); balogh.peter@pte.hu (P.B.); berki.timea@pte.hu (T.B.); nemeth.peter@pte.hu (P.N.); 2 Lymphoid Organogenesis Research Group, Szentagothai Research Center, University of Pecs, H-7624 Pécs, Hungary

**Keywords:** natural autoantibody, pathological autoantibody, autoimmune disease

## Abstract

The natural autoantibody (natAAb) network is thought to play a role in immune regulation. These IgM antibodies react with evolutionary conserved antigens; however, they do not lead to pathological tissue destruction as opposed to pathological autoantibodies (pathAAb). The exact relation between the natAAbs and pathAAbs is still not completely understood; therefore, in the present study, we set out to measure nat- and pathAAb levels against three conserved antigens in a spontaneous autoimmune disease model: the NZB mouse strain which develops autoimmune hemolytic anemia (AIHA) from six months of age. There was an age dependent increase in the natAAb levels in the serum against Hsp60, Hsp70, and the mitochondrial citrate synthase until 6–9 months of age, followed by a gradual decrease. The pathological autoantibodies appeared after six months of age, which corresponded with the appearance of the autoimmune disease. The changes in nat/pathAAb levels were coupled with decreasing B1- and increasing plasma cell and memory B cell percentages. Based on this, we propose that there is a switch from natAAbs towards pathAAbs in aged NZB mice.

## 1. Introduction

The most important function of the immune system is distinguishing self- from non-self (foreign) structures, thereby providing a physiological balance of tolerance- and elimination-type responses. Although in the past decades our knowledge has substantially increased about tolerance mechanisms, some aspects still remain obscure. A complex network of cellular and molecular mechanisms is responsible for self-tolerance, starting from the rigorous selection processes of the T- and B lymphocyte precursors which avoid the exit of autoreactive cells from the primary lymphatic organs, complemented by the Tregs and Bregs, and tolerogenic dendritic cells (DCs) in the periphery [1]. On the molecular side, suppressive cytokines such as IL-10 and TGFβ are key elements, and work together with the natural (auto)antibody network [2].

Earlier, autoantibodies were thought to be the hallmark of autoimmune diseases. However, our knowledge on autoantibodies has changed fundamentally due to the recognition of the natural/physiological IgM autoantibodies which are found in healthy individuals without prior immunization and do not cause pathological tissue damage [3]. They react with a number of genetically and evolutionarily conserved antigens (for example heat shock proteins, cytoskeleton components, cell nuclear structures, mitochondrial enzymes, serum components, etc.); based on this, the “immunological homunculus” or “immunculus” hypothesis was suggested, implying that the network of these natural autoantibodies might play an important role in immune regulation [4]. Despite increasing knowledge about natural autoantibodies, there is still no direct evidence whether changes in their composition or concentration might play a role in the development of autoimmune diseases.

Special B cell subsets such as the B1- and marginal zone (MZ) B cells are thought to be the source of low-affinity polyreactive antibodies, mainly those of the IgM isotype, termed natural antibodies (natAbs) [4,5,6]. In mice, B1 cells mainly reside in pleural- and peritoneal cavities, and are responsible for the production of approximately 80% of IgM antibodies [7,8]. B1 cells are long-lived and have a self-renewing capacity, and they are subdivided into B1a and B1b subsets based on their CD5 expression [3,5,6]. Upon stimulation, B1 cells are able to migrate from peritoneal cavities to the spleen and lymph nodes, and subsequently differentiate into natAb IgM-secreting cells [4,7]. NatAbs are involved in multiple immunological functions such as the initiation of apoptosis, complement activation, FcR-mediated activation, antigen opsonisation, and allograft rejection [4].

A substantial part of natAbs is directed against self-antigens, and these antibodies are called natural autoantibodies (natAAbs) [7,9]. NatAAbs may interact with altered self-antigens and neo-antigens derived from senescent, apoptotic, and necrotic cells, facilitating their removal by phagocytosis [4,9]. Therefore, natAAbs play important roles in tissue homeostasis, and they are essential in protection from the development of autoimmune diseases [7,8,10]. Several studies reported that mice deficient in serum IgM have an increased level of pathogenic IgG autoantibodies [7,11,12,13].

Mouse models are essential tools in studying autoimmune diseases. Similarities of these animal models to humans provide insights in understanding the disease’s pathogenesis, and allow the testing of the safety and efficacy of candidate therapies [5,6]. Spontaneous autoimmune mouse models, for example, the NZB strain, may develop autoimmune hemolytic anemia (AIHA) at various frequencies from the age of six months [7], while the first generation (F1) after crossing with the NZW strain (BW/F1) tend to develop an SLE-like condition between the ages of six and nine months, accompanied with a decreased level of IgM and an increase in anti-DNA IgG [8,10]. Interestingly, some NZB mice after 12 months of age developed a splenic lymphoma, too.

In this study, we characterized the age-dependent changes of the serum natural (nat) and pathological (path) autoantibody (AAb) levels in NZB mice, a model for autoimmune hemolytic anemia. We measured the serum natIgM against evolutionarily conserved antigens: Hsp60 [9], Hsp70 [11], and the mitochondrial citrate synthase (CS) [12]. We show that the level of natIgM AAbs changed in the sera of NZB mice with age and there was a shift towards pathological autoantibodies from the age of 6–9 months. These results confirm that the natural antibody network might indeed play a role in the regulation of autoimmunity.

## 2. Results

### 2.1. Pathological Autoantibody Levels Correlate with the Development of Autoimmune Hemolytic Anemia

**Figure 1:**
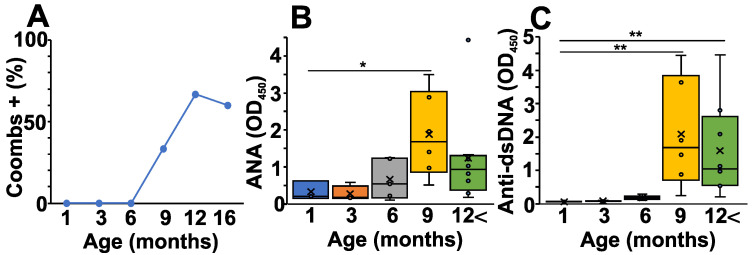
Age-related changes in the pathological autoantibody levels of NZB mice. (**A**) Anti-RBC Ab levels were determined with Coombs test using the sera from NZB mice at different ages. Plot shows the percentage of Coombs positive sera in each age group. (**B**) ANA levels were determined at different ages from the sera of NZB mice (1 month (blue) *n* = 5, 3 months (orange) *n* = 4, 6 months (grey) *n* = 5, 9 months *n* = 6 (yellow), >12 months *n* = 8 (green)). (**C**) Anti-dsDNA antibody levels were determined at different ages from the sera of NZB mice (1 month (blue) *n* = 5, 3 months (orange) *n* = 4, 6 months (grey) *n* = 5, 9 months *n* = 6 (yellow), >12 months *n* = 8 (green)). The box (representing the 25–75% interquartile range wherein the median and average values are indicated with a horizontal line and “x”, respectively) and whiskers (representing the minimum/maximum values) plots show the optical density (O.D.) data from different age groups. Statistically significant differences (Mann–Whitney test) are indicated (* *p* < 0.05, ** *p* < 0.01).

From five to six months of age, NZB mice start to develop AIHA. To follow the characteristic laboratory serum marker for this disease, first, we measured serum anti-RBC antibodies (Coombs test) from the different age groups of NZB mice (Figure 1A). As expected, the percentage of Coombs positive mice was elevated markedly after six months of age, corresponding to the time when pathological autoimmunity develops (Figure 1A). In addition, we also measured the ANA levels, another potential biomarker for systemic autoimmunity (Figure 1B). Similarly to the Coombs test, the ANA levels rose from six months of age (Figure 1B). Finally, we also measured the anti-dsDNA—specific autoantibody levels which showed a somewhat delayed but significant elevation in mice from nine months of age (Figure 1C).

### 2.2. Age-Dependent Changes in the Natural Autoantibody Levels of NZB Mice

**Figure 2:**
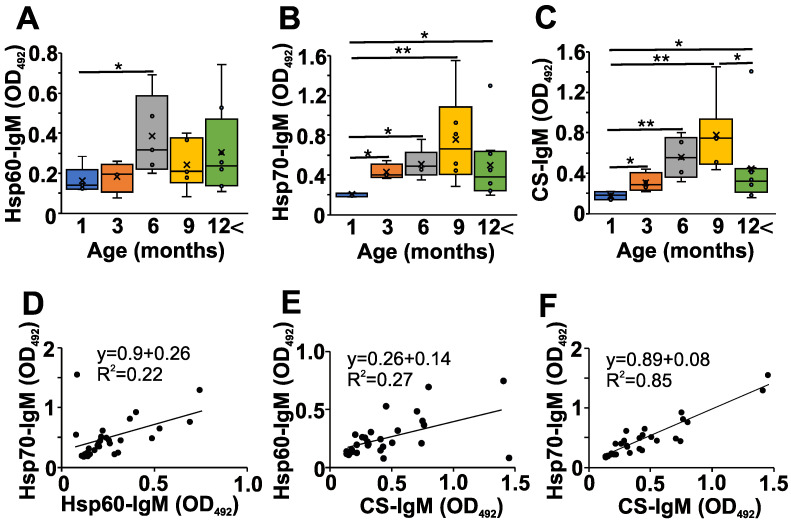
Age-dependent changes in the natAAb levels in the sera of NZB mice. NZB mice at different ages (1 month (blue) *n* = 5, 3 months (orange) *n* = 4, 6 months (grey) *n* = 5, 9 months *n* = 6 yellow), >12 months *n* = 8 (green)) were sacrificed, their sera were collected, and the natural IgM levels against Hsp60 (**A**), Hsp70 (**B**), and CS (**C**) were measured with indirect ELISA. The box (representing the 25–75% interquartile range wherein the median and average values are indicated with a horizontal line and “x”, respectively) and whiskers (representing the minimum/maximum values) plots show the optical density (O.D.) data from different age groups. Statistically significant differences (Mann–Whitney test) are indicated (* *p* < 0.05, ** *p* < 0.01). Scatter plots show the correlations for the Hsp-60 vs. Hsp70 (**D**), CS vs. Hsp60 (**E**) or CS vs. Hsp70 IgM (**F**) O.D. values. Mathematical formulas describing the correlations are indicated on each panel.

Next, we addressed the question of whether changes in the natAAb network might be present during the development of autoimmune disease in NZB mice; so, we measured some serum natIgM levels (Figure 2). We followed the changes in the natAAb levels against Hsp60, Hsp70, and CS at different ages (Figure 2A,B and C, respectively). We chose these three autoantigens because they have already been shown to be recognized by natAAbs [9,11,12]. The levels of serum natIgM Abs against the three analyzed conserved autoantigens were lowest in the one-month-old NZB mice, showing a gradual significant increase up to 6–9 months of age, followed by a marked (but not significant) decrease in older mice (Figure 2A–C).

Since the natIgM autoantibodies are thought to form a network [4] in the serum, and we have seen similar age-related changes in the levels of natIgM against the three different antigens in the NZB strain (Figure 2A–C), we next analyzed the correlation among the levels of individual-specific natIgM antibodies (Figure 2D–F, Table 1). We found significant strong (R > 0.4) positive correlations among the natIgM levels against Hsp60, Hsp70, or the CS antigens in the sera of NZB mice (Figure 2D–F, Table 1).

Correlation analysis of the natIgM and pathAAb levels showed a significant positive correlation between the ANA levels and natIgM reacting with Hsp60, Hsp70, and CS (Table 2). We also found significant positive correlations between the anti-dsDNA and the CS natIgM levels. In contrast, Coombs positivity did not show such s strong correlation with the natIgM levels (Table 2). As expected, the correlation of ANA and anti-dsDNA levels was strong and significant (Table 2).

### 2.3. Age-Dependent Changes of B Cell Composition in the Spleen of NZB Mice

**Figure 3:**
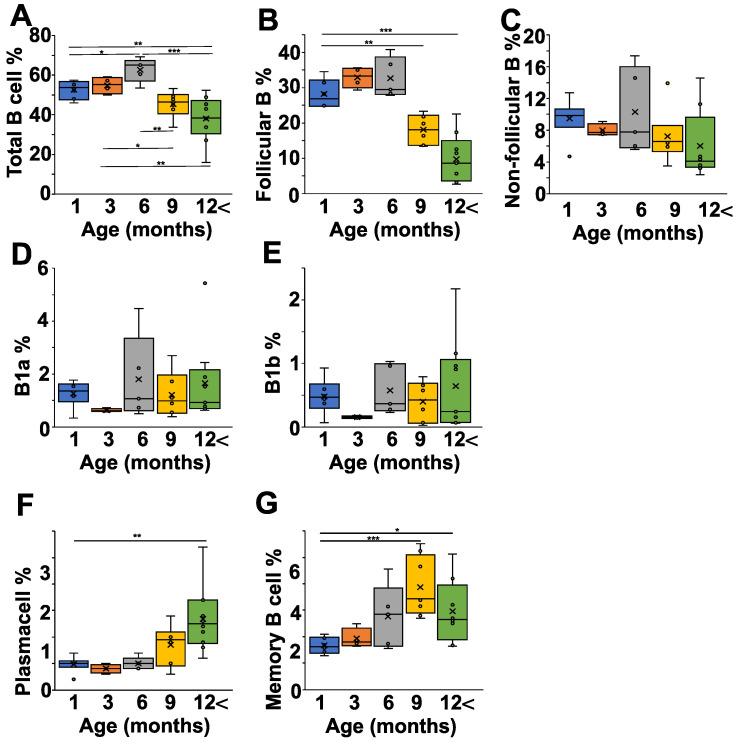
Age-dependent changes of the B cell subpopulations in the spleen of NZB mice. NZB mice at different ages (1 month (blue) *n* = 5, 3 months (orange) *n* = 4, 6 months (grey) *n* = 5, 9 months *n* = 6 (yellow), >12 months *n* = 8 (green)) were sacrificed and their spleen cell composition was analyzed using flow cytometry. We determined the percentages of total B cells (B220^+^ cells) (**A**), the follicular B cells (IgD^high^IgM^low^CD23^+^) (**B**), the non-follicular B cells (IgD^low^IgM^high^CD23^−^) (**C**), the B1a cells (IgM^high^CD43^+^CD5^+^) (**D**), the B1b (IgM^high^CD43^+^CD5^−^) (**E**), the plasma cells (B220^low^, CD138^+^) (**F**), and the memory B cells (B220^low^, CD38^+^CD73^+^ ) (**G**). Corresponding representative flow cytometry data from each age group are shown on Figure S1. The box (representing the 25–75% interquartile range wherein the median and average values were indicated with a horizontal line and “x”, respectively) and whiskers (representing the minimum/maximum values) plots show the percentages of the cell populations from different age groups. Statistically significant differences (Mann–Whitney test) are indicated (* *p* < 0.05, ** *p* < 0.01, *** *p* < 0.005).

After having seen the age-related changes in the pathological and natural autoantibody levels in the NZB strain, we were curious how the B cell subpopulations correlated with all this. First of all, we analyzed the spleen, since in NZB mice it is suspected to be connected to the development of autoimmune hemolytic disease (Figure 3 and Figure S1). The percentage of B cells increased from one month to six months of age significantly (*p* < 0.05), but started to decline after that, and in mice older than 12 months there were significantly less B cells than in mice between one and six months of age (Figure 3A). A more detailed analysis showed that both the follicular (IgD^high^IgM^low^) and non-follicular (IgD^low^IgM^high^) B cell ratio declined with age; however, the follicular B cell population showed a more prominent drop (Figure 3B,C). The non-follicular (IgD^low^IgM^high^) B cells in the spleen represent a mixture of B1-, marginal zone, and transitional B cells. Since B1 cells are thought to produce natural autoantibodies, we analyzed their ratios in the different age groups, but did not find any significant alterations in either the IgM^high^CD43^+^CD5^+^ B1a- or the IgM^high^CD43^+^CD5^-^ B1b cells (Figure 3D,E). In contrast to the decrease in total B cell percentage, the ratio of plasma cells (Figure 3F) and memory B cells (Figure 3G) increased in older mice.

### 2.4. Age-Dependent Changes of B Cell Composition in the Peritoneum of NZB Mice

**Figure 4:**
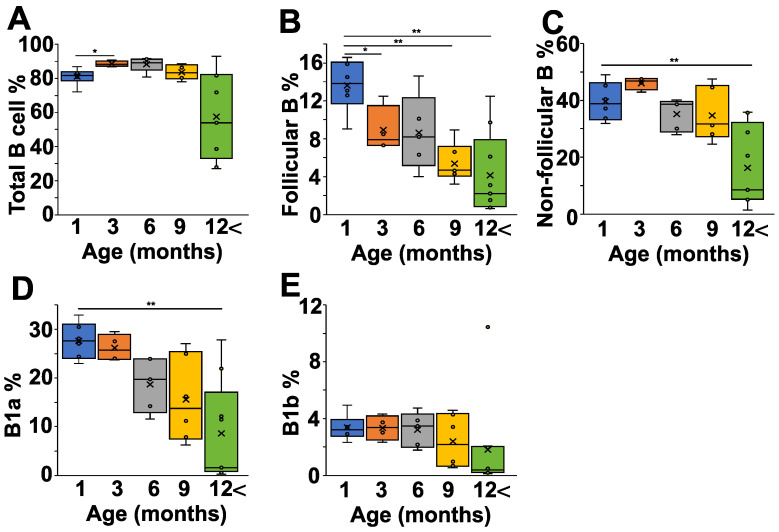
Age-dependent changes of the B cell subpopulations in the peritoneal lavage fluid (PLF) of NZB mice. NZB mice at different ages (1 month (blue) *n* = 5, 3 months (orange) *n* = 4, 6 months (grey) *n* = 5, 9 months *n* = 6 (yellow), >12 months *n* = 8 (green)) were sacrificed and their peritoneal cavity was washed with PBS, and the PLF cell composition was analyzed using flow cytometry. We determined the percentages of total B cells (B220^+^ cells) (**A**), the follicular B cells (IgD^high^IgM^low^CD23^+^) (**B**), the non-follicular B cells (IgD^low^IgM^high^CD23^−^) (**C**), the B1a cells (IgM^high^CD43^+^CD5^+^) (**D**), and the B1b (IgM^high^CD43^+^CD5^−^) cells (**E**). The box (representing the 25–75% interquartile range wherein the median and average values are indicated with a horizontal line and “x”, respectively) and whiskers (representing the minimum/maximum values) plots show the percentages of the cell populations from different age groups. Statistically significant differences (Mann–Whitney test) are indicated (* *p* < 0.05, ** *p* < 0.01).

Finally, we analyzed the peritoneal cells of the NZB mice at different ages, because the peritoneal cavity is thought to be particularly rich in B1 cells. The total B cell percentage showed only minor variations between one and nine months of age, but decreased after 12 months (Figure 4A). We saw a continuous decline in the follicular B cell population with age (Figure 4B), whereas the non-follicular B cell percentage decreased significantly only in mice after 12 months (Figure 4C). A more detailed analysis of the non-follicular population revealed that the IgM^high^CD43^+^CD5^+^ B1a cells showed a continuous decrease with age (Figure 4D), whereas the percentage of B1b cells was more stable over time (Figure 4E).

## 3. Discussion

NatAbs, predominantly of the IgM isotype, are produced by B1 and marginal zone B cells, without immunological stimulation and independently from T cells [13,14,15]. IgM-natAbs provide a crucial early protection against infections before establishing the adaptive immune response [16,17]. IgM-natAbs are always of low-binding affinity and can bind foreign antigens as well as self-antigens [16,18]. The self-antigen-binding IgM-natAbs make up approximately 80% of the total natural antibodies, which can also be referred to as natAAbs [16]. NatAAbs play an important role in the clearance of senescent, apoptotic, and necrotic cells, tumors, and altered self-antigens [16,18,19]. Moreover, IgM-natAbs play a crucial regulatory role in the prevention of autoimmune diseases through avoiding excessive inflammatory immune response, maintaining B cell homeostasis, clearance of damage associated molecular patterns (DAMPs) such as dsDNA, and also the binding of pathogenic IgG autoantibodies as part of their regulatory function [19,20,21,22]. Despite increasing knowledge about natAbs, we are still far from a complete understanding of their potential role in autoimmune disease(s).

In the present study, we measured the natAAb levels against Hsp60, Hsp70, and CS antigens because it has been verified earlier that they are recognized by natAb-s. Hsp60- and Hsp70-specific IgM antibodies were described in human cord blood samples [23]. Interestingly, their elevated levels were reported in type I diabetes [24], arthritis [25], and atherosclerosis [26]. Thus, natAbs recognizing these Hsp molecules are related to both the natural and pathological sides of autoimmunity [27]. The mitochondrial CS was also suggested to be an evolutionarily conserved antigen recognized by natAbs [12]. Moreover, a fine epitope mapping study using phage display revealed that different epitopes of the CS are recognized under physiological and pathological conditions [28].

AIHA is an antibody-mediated uncommon immune disorder, where autoantibodies attack and destroy erythrocytes, leading to uncompensated erythrocytes loss [29,30]. Although they show individual variation, NZB mice are the traditionally studied models for spontaneous AIHA [31]. Although the NZB mouse model has been known for decades, spontaneous AIHA and the following lymphoma development are not yet elucidated, and serum natAb levels have been only partially characterized [32]. In the present study, we report that the serum natAb levels showed changes during aging of NZB mice; first, there was a marked increase in the natAb levels until 6–9 months of age followed by a decrease in older mice. Meanwhile, at around six months of age, the level of pathological autoantibodies started to increase which correlated well with the spontaneous development of the AIHA. Changes in the B cell subpopulations corresponded to changes in the autoantibody levels; the natural autoantibody producing B1 cell percentage decreased in older mice, while the plasma cells and the memory B cells, which could be responsible for pathological autoantibody production, increased in aged mice. Similarly decreased B1 cell percentage in the spleen was also found in BALB/c mice during aging [33]. We propose that the shift from the natural autoantibodies towards the pathological autoantibodies, which occurred after six months of age, could contribute to the NZB phenotype. Moreover, the levels of natAAbs remained relatively high even after six months of age, suggesting their possible compensatory role, but they could not protect the mice from the autoimmune disease.

We found no other study where the natAb levels against Hsp60 or Hsp70 were followed systematically over time either in humans or mice. In case of the CS, IgM-type antibodies showed relatively constant levels in 53 healthy blood donors followed over a five year period and measured at three occasions [12]. However, even this latter report is restricted to a relatively short period [12], when compared to our study here, where we followed the natAb levels from early life (one month of age) to senescence (more than 12 months of age). In a previous study in NZB mice, natAb levels against actin, myosin, myoglobin, tubulin, spectrin, and DNA had been followed at different ages [32], showing similar changes to our results here: a gradual increase over time in the nat IgM levels replaced by IgG after 10–12 months of age.

The F1 hybrids of the NZB and the NZW strain are known for the spontaneous development of an SLE-like pathology. Systemic lupus erythematosus (SLE) is a heterogeneous autoimmune disease characterized by immune complexes deposition, inflammation and tissue damage, and the inefficient clearance of apoptotic cells [34,35,36]. This leads to the production of autoantibodies against cellular and nuclear components, such as antinuclear autoantibodies (ANA) including anti-ds-DNA, anti-Smith, and anti-histone antibodies [8,34,36]. The BW/F1 mice developed a similar pathology from six months of age [37]. Interestingly, we measured elevated levels of the ANA and anti-dsDNA autoantibodies in the older NZB mice, too. Although the NZB mice had a significantly longer life expectancy than the BW/F1 mice based on our own observations (15–16 months vs. 9–11 months, respectively) in some cases they also developed kidney disease, which might be due to this latent lupus-like phenotype.

The age-related variations of the natAAb network in the NZB model presented here draws the attention to the importance of individual natAAb composition in humans. Susceptibility to autoimmune diseases is based on several factors (MHC genes, life-style, infections, microbiota, etc.). Perhaps added to these factors, the natAAb network should also be considered in the future.

## 4. Materials and Methods

### 4.1. Mice and Collection of the Sera

We used female NZB mice (founders purchased from Charles River Germany) at different ages for our studies (Table 3). Mice were kept under SPF conditions at 24 ± 2 °C with a controlled 12/12 h light/dark cycle, at the Department of Immunology and Biotechnology’s Transgenic Mouse Facility. The mice were housed in groups of five and received acidified water and food ad libitum. All animal experiments were conducted following the University of Pécs’s Animal Welfare Committee regulations.

After sacrificing the mice, we collected part of their blood with heparin for Coomb’s test, and the rest without heparin for the isolation of serum which was stored at −80 °C until use.

### 4.2. Antibodies and Reagents

All chemicals were purchased from Sigma-Aldrich unless otherwise stated.

For flow cytometry we used the following: washing buffer, PBS containing 0.1% NaN_3_; staining buffer, PBS containing 0.1% BSA and 0.1% NaN_3_; and fixation buffer, PBS containing 0.1% paraformaldehyde [38].

For ELISA we used the following: 0.1 M Na_2_CO_3_/NaHCO_3_ carbonate (pH: 9.4) coating buffer; PBS containing 1% non-fat dry milk blocking buffer; and PBS containing 0.1% Tween-20 washing buffer [38].

The following monoclonal antibodies were used for flow cytometry: anti-CD38-PE, anti-CD73-Alexa Fluor 647, anti-IgD-FITC, anti-IgM-PerCP-Cy5.5, anti-CD138-APC-R700, anti-B220-PE-Cy7, and CD23-BV421, all from BD Bioscience (San Jose, CA, USA). For ELISA, HRP rat anti-mouse IgM (BD Bioscience) was used [38].

### 4.3. Coombs Test

For the direct Coomb’s test [39], we collected the blood in tubes containing 100 μL of heparin. The samples were kept at RT for at least 4 h. We centrifuged the samples for 10 min on 4 °C with 1000 *g*. The supernatant was removed and the red blood cells (RBC) were washed in 750 μL of PBS containing 1% BSA 3 times. The RBC pellet was resuspended in PBS containing 1% BSA at a 100-fold dilution. Goat anti-mouse IgG 2a antibody was diluted 40-fold in PBS containing 0.1% BSA and 0.1% NaN_3_. Equal volumes (50 μL) from the RBC suspension and the antibody solution were mixed in a 96-well U-shaped bottom test plate per well and incubated for one hour at RT in duplicates. Finally, the wells were evaluated for agglutination and were photodocumented.

### 4.4. ELISA

We measured the serum natural autoantibody IgM levels against Hsp60, Hsp70, and CS using indirect ELISA [12,40]. Briefly, we coated Nunc Maxisorb ELISA plates with 5 μg of recombinant Hsp60, -Hsp70, or citrate-synthase in carbonate coating buffer overnight at 4 °C. After the coating, plates were washed 4 times and blocked for 1 h at RT followed by washing 4 times again. The sera were added at a 1:100 dilution and incubated for 2 h at 37 °C. Next, we washed the plates 4 times, before adding the secondary PO-conjugated anti-mouse IgM at a 1:1000 dilution in PBS and incubated for 1 h at RT. The color reaction was developed with the addition of OPD substrate solution, which was stopped with sulfuric acid after 20 min. Optical density values were read at 492 nm with the iEMS reader (MF Thermo Labsystems, Philadelphia, PA, USA).

ANA antibodies were measured using the QANTA Lite ELISA Kit (Inova Diagnostics, San Diego, CA, USA) according to the manufacturer’s instructions, with slight modifications. The reaction was developed using peroxidase-conjugated anti-mouse-IgG1 (BD Bioscience, San Jose, CA, USA) as secondary antibodies [38].

### 4.5. Flow Cytometry

After sacrifice, we rinsed the peritoneal cavity of the mice with ice cold PBS and then collected the spleens. Peritoneal lavage fluid (PLF) was centrifuged to collect the cells, the spleens were homogenized using a mesh and hemolyzed. Briefly, 10^6^ cells/sample were washed twice with flow cytometry washing buffer and then incubated with different cocktails of fluorochrome-conjugated monoclonal antibodies diluted in flow cytometry staining buffer for 30 min, at RT in the dark. Finally, the samples were washed twice and resuspended in a flow cytometry fixation buffer. Data acquisition was performed using a FACS Canto II flow cytometer and FACS DIVA software (Version 6.1.3, BD Biosciences) for data analysis. We defined the following cell subsets based on surface markers. B220^+^: B cells; IgD^high^IgM^low^CD23^+^: follicular B cells; IgD^low^IgM^high^CD23^−^: non-follicular (B1- and marginal zone) B cells; IgM^high^CD43^+^CD5^+^: B1a cells; IgM^high^CD43^+^CD5^−^: B1b cells; B220^low^CD73^+^CD38^+^: memory B cells; B220^low^CD138^+^: plasma cells [38].

### 4.6. Statistical Analyisis

Data analysis was performed using MS Excel version 16.72 and GraphPad Prism version 5.03 software. Data in the box and whiskers plots are presented as the 25–75% interquartile range (the median and average values are indicated with a horizontal line and “x”, respectively) and the minimum/maximum values, respectively. An ANOVA test was used to compare the experimental groups using Dunnett’s post hoc test with unequal variances, and *p*-values ≤ 0.05 were considered statistically significant. For correlation analyses, Pearson’s correlation coefficients (R) were calculated, where *p*-values ≤ 0.05 were considered statistically significant. For comparing the two groups, the Mann–Whitney test was used, and *p*-values ≤ 0.05 were considered statistically significant.

## Figures and Tables

**Table 1:** Correlations among the measured natIgM Ab levels in the sera of NZB mice.

	Hsp60	Hsp70	CS
**Hsp60**	1	0.465 *	0.521 **
**Hsp70**	0.465 *	1	0.923 **
**CS**	0.521 **	0.923 **	1

Values show the Pearson’s correlation coefficient (R) calculated based on the O.D. data of serum natural IgM antibody levels. R > 0 or R < 0 indicate positive or negative correlations, R values closer to 1 or −1 indicate a stronger correlation. Significant correlations are indicated (* *p* ≤ 0.05 or ** *p* ≤ 0.01). Corresponding correlation scatter plots are shown in Figure S1. The antigens recognized by natural IgM antibodies are bold.

**Table 2:** Correlations among the measured natIgM and the pathological Ab levels in the sera of NZB mice.

	Hsp60	Hsp70	CS	ANA	Anti-dsDNA	Coombs
**ANA**	0.466 *	0.563 *	0.667 **	1	0.689 **	0.125
**anti-dsDNA**	0.226	0.281	0.472 *	0.689 **	1	0.151
**Coombs**	−0.012	0.205	0.151	0.125	0.151	1

Values show the Pearson’s correlation coefficient (R) calculated based on the O.D. data of serum natural IgM antibody levels. Note, in the case of the Coombs test, we converted the positive or negative results into numerical values 1 or 0, respectively. R > 0 or R < 0 indicated positive or negative correlation, and R values closer to 1 or −1 indicated a stronger correlation. Significant correlations are indicated (* *p* ≤ 0.05 or ** *p* ≤ 0.01). The antigens recognized by natural- and pathological antibodies are bold.

**Table 3:** Experimental mouse groups used in the study.

Age Range ^1^	Average Age ^1^	Number
1–1.1	1.04	5
3–3.3	3.2	4
6–6.7	6.14	5
8.6–9.4	9	6
11.6–17.2	14	8

^1^ Age range and average age values are expressed in months.

## Data Availability

Not applicable.

## Supplementary Materials

The supporting information can be downloaded at https://www.mdpi.com/article/10.3390/ijms24129809/s1.

## References

[1] Abbas A., Lichtman A., Pillai S. (2010). Cellular and Molecular Immunology.

[2] Cohen I.R. (1992). The cognitive paradigm and the immunological homunculus. Immunol. Today.

[3] Lacroix-Desmazes S., Kaveri S.V., Mouthon L., Ayouba A., Malanchère E., Coutinho A., Kazatchkine M.D. (1998). Self-reactive antibodies (natural autoantibodies) in healthy individuals. J. Immunol. Methods.

[4] Cohen I.R., Young D.B. (1991). Autoimmunity, microbial immunity and the immunological homunculus. Immunol. Today.

[5] Lee B.H., Gauna A.E., Pauley K.M., Park Y.J., Cha S. (2012). Animal models in autoimmune diseases: Lessons learned from mouse models for Sjögren’s Syndrome. Clin. Rev. Allergy Immunol..

[6] Yu X., Petersen F. (2018). A methodological review of induced animal models of autoimmune diseases. Autoimmun. Rev..

[7] Howie H.L., Hudson K.E. (2018). Murine models of autoimmune hemolytic anemia. Curr. Opin. Hematol..

[8] Karnopp T.E., Chapacais G.F., Freitas E.C., Monticielo O.A. (2021). Lupus animal models and neuropsychiatric implications. Clin. Rheumatol..

[9] Feige U., van Eden W. (1996). Infection, autoimmunity and autoimmune disease. Stress-Inducible Cell. Responses.

[10] Hirose S., Kinoshita K., Nozawa S., Nishimura H., Shirai T. (1990). Effects of major histocompatibility complex on autoimmune disease of H-2-congenic New Zealand mice. Int. Immunol..

[11] Radons J. (2016). The human HSP70 family of chaperones: Where do we stand?. Cell Stress Chaperones.

[12] Czömpöly T., Olasz K., Simon D., Nyárády Z., Pálinkás L., Czirják L., Berki T., Németh P. (2006). A possible new bridge between innate and adaptive immunity: Are the anti-mitochondrial citrate synthase autoantibodies components of the natural antibody network?. Mol. Immunol..

[13] Kappler K., Hennet T. (2020). Emergence and significance of carbohydrate-specific antibodies. Genes Immun..

[14] Schwartz-Albiez R., Monteiro R.C., Rodriguez M., Binder C.J., Shoenfeld Y. (2009). Natural antibodies, intravenous immunoglobulin and their role in autoimmunity, cancer and inflammation. Clin. Exp. Immunol..

[15] Palma J., Tokarz-Deptuła B., Deptuła J., Deptuła W. (2018). Natural antibodies—Facts known and unknown. Cent. Eur. J. Immunol..

[16] Reyneveld G.I.J., Savelkoul H.F.J., Parmentier H.K. (2020). Current Understanding of Natural Antibodies and Exploring the Possibilities of Modulation Using Veterinary Models. A Review. Front. Immunol..

[17] Xu X., Ng S.M., Hassouna E., Warrington A., Oh S.H., Rodriguez M. (2015). Human-derived natural antibodies: Biomarkers and potential therapeutics. Future Neurol..

[18] Lobo P.I. (2016). Role of natural autoantibodies and natural IgM anti-leucocyte autoantibodies in health and disease. Front. Immunol..

[19] Panda S., Ding J.L. (2015). Natural Antibodies Bridge Innate and Adaptive Immunity. J. Immunol..

[20] Notley C.A., Baker N., Ehrenstein M.R. (2010). Secreted IgM Enhances B Cell Receptor Signaling and Promotes Splenic but Impairs Peritoneal B Cell Survival. J. Immunol..

[21] Ehrenstein B.M.R., Cook H.T., Neuberger M.S. (2000). Deficiency in Serum Immunoglobulin (Ig) M Predisposes to Development of IgG Autoantibodies. J. Exp. Med..

[22] Boes M., Schmidt T., Linkemann K., Beaudette B.C., Marshak-rothstein A., Chen J. (2000). Accelerated development of IgG autoantibodies and autoimmune disease in the absence of secreted IgM. Proc. Natl. Acad. Sci. USA.

[23] Merbl Y., Zucker-Toledano M., Quintana F.J., Cohen I.R. (2007). Newborn humans manifest autoantibodies to defined self molecules detected by antigen microarray informatics. J. Clin. Investig..

[24] Elias D., Reshef T., Birk O.S., Van Der Zee R., Walker M.D., Cohen I.R. (1991). Vaccination against autoimmune mouse diabetes with a T-cell epitope of the human 65-kDa heat shock protein. Proc. Natl. Acad. Sci. USA.

[25] Quintana F.J., Carmi P., Mor F., Cohen I.R. (2004). Inhibition of adjuvant-induced arthritis by DNA vaccination with the 70-kd or the 90-kd human heat-shock protein: Immune cross-regulation with the 60-kd heat-shock protein. Arthritis Rheum..

[26] Almanzar G., Öllinger R., Leuenberger J., Onestingel E., Rantner B., Zehm S., Cardini B., van der Zee R., Grundtman C., Wick G. (2012). Autoreactive HSP60 epitope-specific T-cells in early human atherosclerotic lesions. J. Autoimmun..

[27] Cohen I.R. (2013). Autoantibody repertoires, natural biomarkers, and system controllers. Trends Immunol..

[28] Czömpöly T., Olasz K., Nyárády Z., Simon D., Bovári J., Németh P. (2008). Detailed analyses of antibodies recognizing mitochondrial antigens suggest similar or identical mechanism for production of natural antibodies and natural autoantibodies. Autoimmun. Rev..

[29] Merashli M., Arcaro A., Graf M., Gentile F., Ames P.R.J. (2021). Autoimmune haemolytic anaemia and antiphospholipid antibodies in paediatrics: A systematic review and meta-analysis. Clin. Rheumatol..

[30] Barcellini W., Fattizzo B. (2020). The Changing Landscape of Autoimmune Hemolytic Anemia. Front. Immunol..

[31] Helyer B.J., Howie J.B. (1963). Spontaneous Auto-Immune Disease in NZB/BL Mice. Br. J. Haematol..

[32] Hentati B., Brogard B.P., Jouanne C., Avrameas S., Ternynck T. (1994). Natural Autoantibodies are Involved in the Haemolytic Anaemia of NZB Mice. J. Autoimmun..

[33] Tarjanyi O., Boldizsar F., Nemeth P., Mikecz K., Glant T.T. (2009). Age-related changes in arthritis susceptibility and severity in a murine model of rheumatoid arthritis. Immun. Ageing.

[34] Bolouri N., Akhtari M., Farhadi E., Mansouri R., Faezi S.T., Jamshidi A., Mahmoudi M. (2022). Role of the innate and adaptive immune responses in the pathogenesis of systemic lupus erythematosus. Inflamm. Res..

[35] Chen P.M., Tsokos G.C. (2022). Mitochondria in the Pathogenesis of Systemic Lupus Erythematosus. Curr. Rheumatol. Rep..

[36] Böröcz K., Simon D., Erdő-Bonyár S., Kovács K.T., Tuba, Czirják L., Németh P., Berki T. (2021). Relationship between natural and infection-induced antibodies in systemic autoimmune diseases (SAD): SLE, SSc and RA. Clin. Exp. Immunol..

[37] Andrews B.S., Eisenberg R.A., Theofilopoulos A.N., Izui S., Wilson C.B., McConahey P.J., Murphy E.D., Roths J.B., Dixon F.J. (1978). Spontaneous murine lupus-like syndromes. Clinical and immunopathological manifestations in several strains. J. Exp. Med..

[38] Khanfar E., Olasz K., Gajdócsi E., Jia X., Berki T., Balogh P., Boldizsár F. (2022). Splenectomy modulates the immune response but does not prevent joint inflammation in a mouse model of RA. Clin. Exp. Immunol..

[39] Coombs R.R.A., Mourant A.E., Race R.R. (1945). A new test for the detection of weak and incomplete Rh agglutinins. Br. J. Exp. Pathol..

[40] Böröcz K., Kinyó Á., Simon D., Erdő-Bonyár S., Németh P., Berki T. (2023). Complexity of the Immune Response Elicited by Different COVID-19 Vaccines, in the Light of Natural Autoantibodies and Immunomodulatory Therapies. Int. J. Mol. Sci..

