# Supplementary material for: Natural and Pathological Autoantibodies Show Age-Related Changes in a Spontaneous Autoimmune Mouse (NZB) Model
Source: Int J Mol Sci. 2023 Jun 6;24(12):9809. doi: 10.3390/ijms24129809 (PMC10298727; doi:10.3390/ijms24129809)
Supplement: Supplementary file 1 [file ijms-24-09809-s001.zip › ijms-2415151-supplementary.pdf]

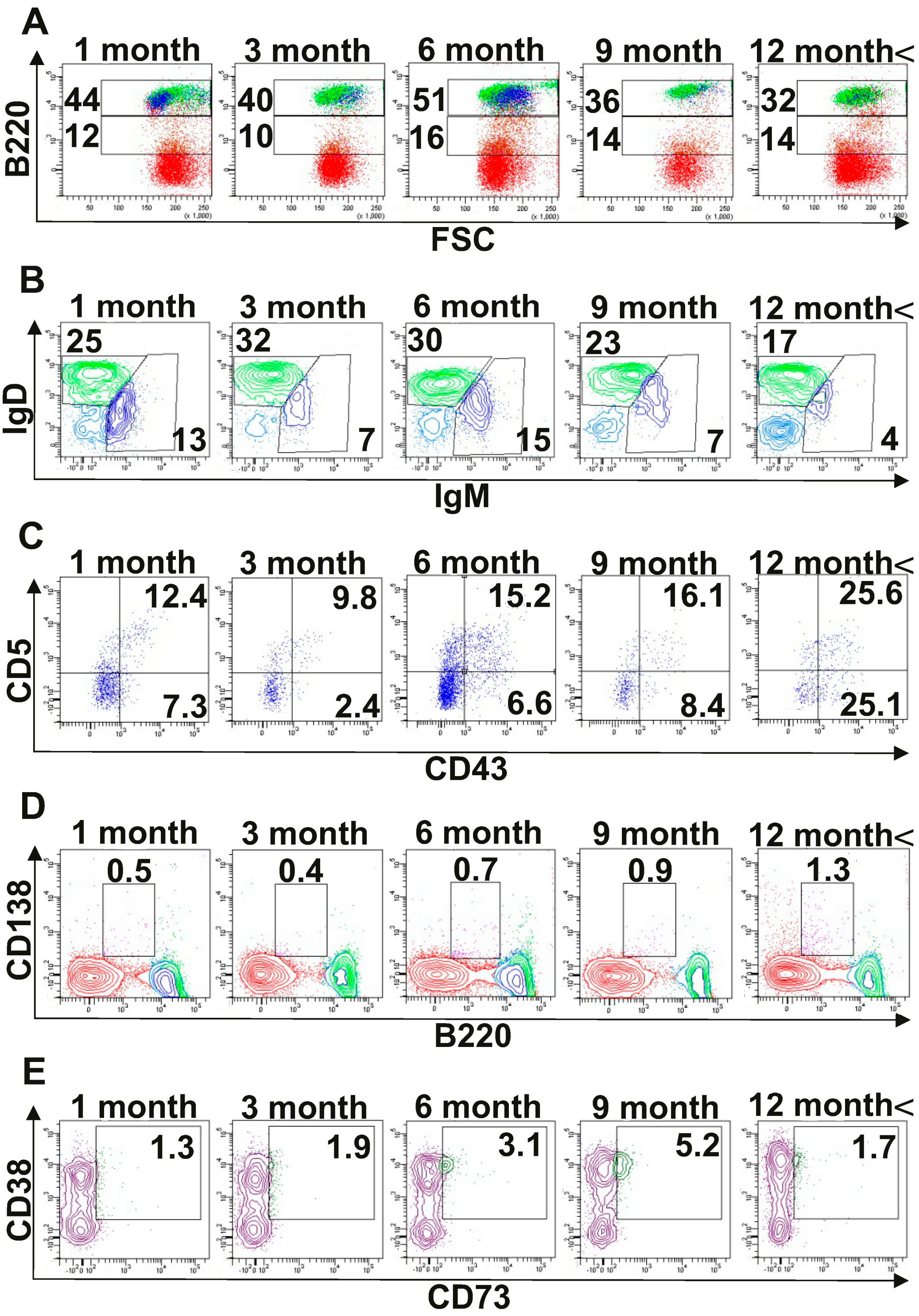

Figure S1: Flow cytometric analysis of the spleen cells isolated from NZB mice at different ages. **A:** Representative flow cytometric dot-plots show the distribution of cells based on their anti-B220 staining and FSC parameters. The numbers in the plots show the percentages of B220^{high} and B220^{low} cells, respectively. **B:** Representative contour plots show the distribution of cells based on their anti-IgD and anti-IgM staining. The numbers in the plots show the percentages of follicular- (IgD^{high}IgM^{low}) and non-follicular (B1- and MZ B cells, IgD^{low}IgM^{high}) B cells indicated with green and dark blue colors, respectively. **C:** Representative dot plots show the distribution of cells based on their anti-CD5 and anti-CD43 staining. The numbers in the plots show the percentages of B1a- (IgM^{high}CD43^{+}CD5^{+}, upper right quadrant) and B1b cells (IgM^{high}CD43^{+}CD5^{-}, lower right quadrant),

respectively. **D:** Representative contour plots show the distribution of cells based on their anti-CD138 and anti-B220 staining. The numbers in the plots show the percentages of plasmacells ($B220^{low}CD138^{+}$). **E:** Representative contour plots show the distribution of $B220^{low}$ cells (see panels **A**) based on their anti-CD38 and anti-CD73 staining. The numbers in the plots show the percentages of memory B cells ($CD38^{+}CD73^{+}$).
